# P-850. Creation and Implementation of a Clinician-Level Outpatient Antimicrobial Stewardship Dashboard

**DOI:** 10.1093/ofid/ofaf695.1058

**Published:** 2026-01-11

**Authors:** Lauren McDaniel, Elizabeth Nowak, Mandy Swann, Ryan Fulton, Nicholas Stornelli, Nathan Everson, Anthony Baffoe-Bonnie

**Affiliations:** Carilion Clinic, Roanoke, VA; Carilion Clinic, Roanoke, VA; Carilion Clinic, Roanoke, VA; Carilion Childrens, Daleville, Virginia; Carilion Clinic, Roanoke, VA; Henry Ford Hospital, Detroit, MI; Carilion Clinic, Roanoke, VA

## Abstract

**Background:**

Tracking and Reporting are Core Elements of effective outpatient antimicrobial stewardship, as outlined by the CDC. While clinician-level feedback is ideal, it is often difficult to operationalize. We developed and implemented the *Antimicrobial Footprint*, a clinician-facing dashboard, to provide individualized antibiotic prescribing feedback and identify opportunities for prescribing optimization across a large health system.

**Figure 1.**
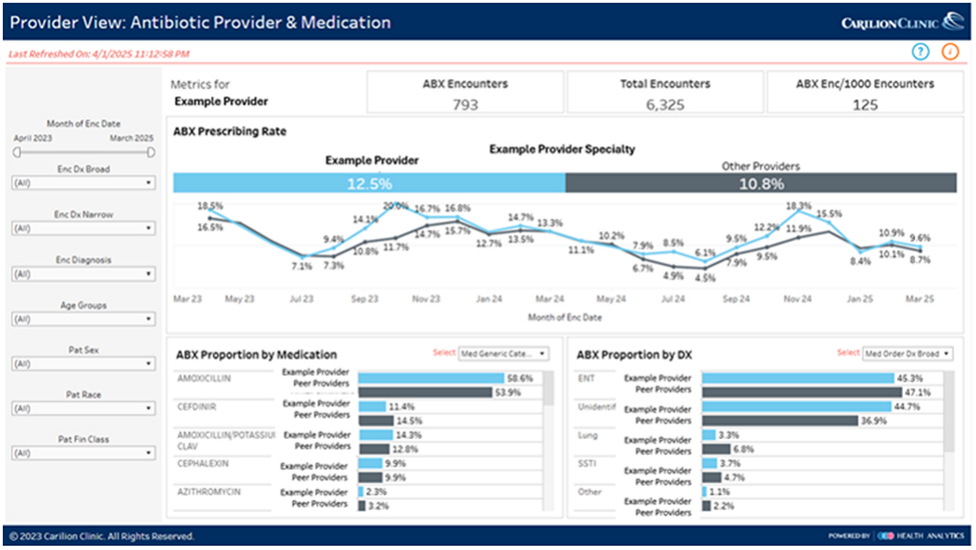
Example of the Antimicrobial Footprint Dashboard

**Figure 2.**
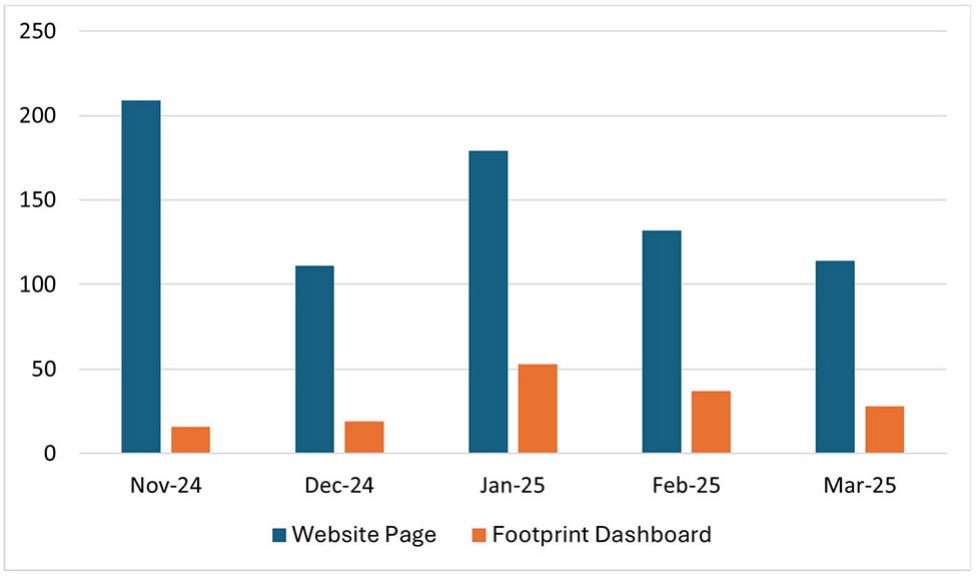
Views to the dashboard website vs. provider clicks into their individualized footprint dashboard

**Methods:**

The dashboard data consisted of ambulatory encounters, including virtual, telephonic and in-person visits. Encounters with an oral systemically administered antibiotic were included. The individualized dashboard allowed providers to see their own prescribing rates compared to aggregate rates of other providers in their specialty. The initial filters included encounter diagnosis, age groups, patient sex, patient race and financial class. Providers could also filter by specific medications. The dashboard development required multiple iterations and technical support for 24 months (Sept 2022-Sept 2024). It had a soft launch in November 2024 during Antibiotic Awareness Week and officially launched in January 2025 (Figure 1). We describe the experience of dashboard utilization post-implementation.

**Table 1.**
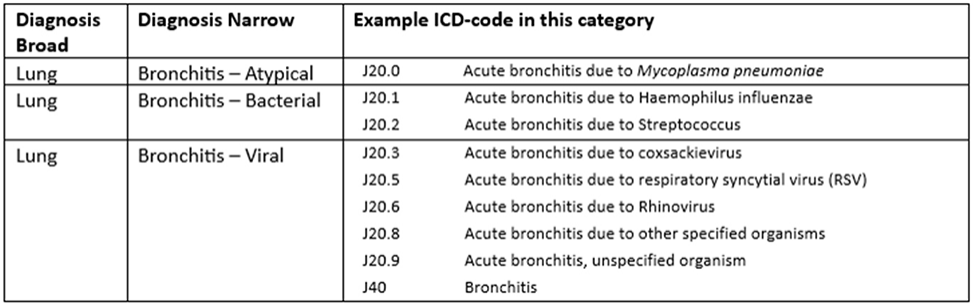
Example diagnosis codes recategorization

**Results:**

In the first five months post-launch, the website hosting the dashboard and additional resources including tutorial videos of using the dashboard, received higher views than the individual clinician dashboard (Figure 2). The clinician tutorial videos received 63 views. Early clinician feedback identified limitations in diagnoses code filters prompting refinement. For example, bronchitis categories were expanded into atypical, bacterial, and viral etiologies (Table 1) to better contextualize prescribing appropriateness.

**Conclusion:**

Developing a clinician-specific outpatient antimicrobial dashboard is feasible and provides valuable insights into prescribing behaviors. However, passive availability alone was insufficient to drive widespread clinician engagement. Future directions include proactive dissemination strategies, integrating dashboard data into routine reporting, and continued refinement to maximize impact.

**Disclosures:**

Ryan Fulton, DO, Microsoft: Advisor/Consultant

